# Editorial: Nanozymes: From Rational Design to Biomedical Applications

**DOI:** 10.3389/fchem.2021.670767

**Published:** 2021-03-30

**Authors:** Kelong Fan, Youhui Lin, Vipul Bansal

**Affiliations:** ^1^Chinese Academy of Sciences (CAS) Engineering Laboratory for Nanozyme, Key Laboratory of Protein and Peptide Pharmaceutical, Institute of Biophysics, Chinese Academy of Sciences, Beijing, China; ^2^Nanozyme Medical Center, School of Basic Medical Sciences, Zhengzhou University, Zhengzhou, China; ^3^Fujian Provincial Key Laboratory for Soft Functional Materials Research, Department of Physics, Research Institute for Biomimetics and Soft Matter, Xiamen University, Xiamen, China; ^4^Ian Potter NanoBioSensing Facility, NanoBiotechnology Research Laboratory, School of Science, Royal Melbourne Institute of Technology (RMIT) University, Melbourne, VIC, Australia

**Keywords:** nanozyme, enzymatic activities, biosensor, antioxidant, catalytic therapy

As the next generation of artificial enzymes, nanozymes are functional nanomaterials that are able to mimic the catalytic function of enzymes. Like natural enzymes, nanozymes can effectively catalyze the conversion of enzyme substrates under mild conditions and exhibit similar catalytic efficiencies and reaction kinetics. Moreover, in certain applications, compared with natural enzymes, nanozymes show several advantages, including multifunctionality, tunability of catalytic activities, low cost, scalable production, and high stability. So far, more than 900 nanozymes have been reported from 350 laboratories in 30 countries, and their applications have been extended to the fields of biology, medicine, agriculture, and environmental governance. In this special issue, we compiled 13 papers from 72 authors on the latest advances in nanozymes and their applications.

As an important advance in the field, the development of nanozyme-based probes for sensing important targets has become one of the hot topics in this research area. For example, the trimetallic PdCuAu nanozyme (Nie et al.) was used as a difunctional probe for temperature monitoring and molecular detection. The thermosensitive property of PdCuAu nanozyme was used for temperature sensing, and the peroxidase-like activity was applied to detect H_2_O_2_ and glucose. In another work, Long et al. decorated Au@Pt@Mesoporous SiO_2_ nanozyme with antigens to detect mumps virus. The core–shell structure with Au@Pt nanozymes and mesoporous SiO_2_ guaranteed the interaction of Au@Pt nanozymes and substrates, as well as prevented antigen molecules from blocking the active nanozyme core. In another study, Nguyen et al. designed a nanozyme-based test strip by utilizing the special color change property of cerium oxide nanozymes. The cerium oxide nanozyme and cholesterol oxidase were immobilized on the strips. When cholesterol was introduced, it was catalyzed by cholesterol oxidase to produce H_2_O_2_, leading to the color change of cerium oxide nanozymes from white to yellow. This work developed a point-of-care testing method for cholesterol without requiring an additional chromogenic substrate. The authors also reported a method to quantify the results of nanozyme-strip by a smartphone.

It has been reported that enzyme-like activities of nanozymes can be selectively regulated by specific molecules. Therefore, many nanozyme-based molecular detection strategies are also developed based on this property. Platinum nanozymes exhibiting robust oxidase-like activity were synthesized and modified using sodium alginate (SA-PtNPs) (He et al.). As an antioxidant, oligomeric proanthocyanidin inhibited the oxidase-like activity of platinum nanozymes, resulting in a reduction of color change intensity. Besides antioxidants, dopamine can suppress the catalytic activities as well. Yang et al. developed a novel method to synthesize Fe_2_O_3_ nanozyme, which was deposited on the surface of carbon nanotubes obtained by atomic layer deposition. By this way, the size of Fe_2_O_3_ nanozymes was precisely controlled within 1 nm, leading to the excellent peroxidase-like activity. When the hybrid nanozymes were used to detect dopamine, the limit of detection was as low as 0.11 μM.

In spite of multiple applications, it is worth noting that only few investigations have focused on understanding the mechanisms of inhibition on the enzymatic activities of nanozymes. In this context, Liang et al. pointed out that the specific complexation between salicylic acid and Fe^3+^ in the active center of MIL-53(Fe) induced the inhibition effect of the peroxidase-like activity. Then the rapid colorimetric sensing platform was constructed with high selectivity and sensitivity to salicylic acid. Apart from that, Mo et al. carried out a detailed study about the inhibition of iron oxide nanozymes (IONzyme) caused by guanidine chloride (GuHCl), a commonly used protein denaturant. In this research, Guanidine chloride disturbed the peroxidase-like activity of IONzymes and resulted in nanoparticle aggregation, which was found as not the foremost reason for the nanozyme inactivation. The results of electron spin resonance spectroscopy revealed a change in unpaired electrons, based on which, the authors attributed the suppression of activity to the interaction of GuHCl with iron atoms. By analyzing the Michaelis-Menten model, they concluded that the GuHCl competed with the substrate H_2_O_2_ and bound to IONzymes.

In addition to a number of original research outputs reported in this special issue, several excellent reviews provide us with a comprehensive understanding of recent progress made in different aspects of nanozymes research. For instance, the intrinsic enzyme-like activity of IONzymes has been exploited for decades. Sutrisno et al. has nicely summarized the previous clinical exploitations of IONzymes for cancer therapy while placing special emphasis on major factors impacting upon their catalytic efficiency and therapeutic performance, as well as outlining representative studies to overcome certain limitations. Yuan et al. highlighted the most recent methods of nano iron sulfide synthesis, including modifications and characterizations. Strikingly, nano-sized iron sulfides demonstrate versatile physicochemical properties, enzyme-like catalysis, high stability and biocompatibility, which facilitates their biomedical applications.

Several other kinds of nanozymes have also been gradually developed. Zhang et al. discussed the enzymatic attributes and biomedical applications of gold clusters. Gold cluster nanozymes possess multiple enzyme-like activities, which are affected by different loading materials, or environment. In regard to applications, ion detection is a common utilization of gold cluster nanozymes, on account of their high susceptibility to heavy metal ions and anions. Further, the luminescence of gold cluster nanozymes is in favor of combing imaging and therapy for *in vivo* theranostics. Finally, challenges including biodistribution and reaction mechanisms of gold nanoclusters need further study. Cai and Yang summarized a wider category of nanozymes—two-dimensional nanomaterials (2D NMs). The top-down and bottom-up methodologies are commonly used methods to synthesize layered and non-layered 2D NMs. The multiple enzyme-like properties are affected by the type of material and the synthesis method. As for application, the authors emphasized the importance of toxicology studies, noting that the biocompatibility of these materials may be related to several structural/compositional parameters and physicochemical properties. Modifying these materials with biocompatible molecules may offer a viable solution for their applications. Tian et al. directed our attention to the rational design of antioxidant nanozymes. Nanozymes with SOD- and catalase-like activities were highlighted as appropriate candidates for antioxidant application. Ways to regulate the antioxidant activities of nanozymes were summarized, and the applications of anti-aging study, inflammation treatment, neurological diseases, and other biological applications were introduced. Huang et al. noticed that inorganic nanomaterials play a role in modulating biomimetic catalytic performance, including those of traditional artificial enzymes and nanozymes. For traditional artificial enzymes, inorganic nanomaterials may interact with active molecules, which may enhance their catalytic efficiency and induce special attractive features. When modulating nanozymes, inorganic nanomaterials regulate the stability and activity of nanozymes and influence multiple catalytic systems. Rational design strategies and in-depth investigation of potential applications are likely to bloom the field of this hybrid nanotechnology.

Despite the remarkable progress made in nanozyme research, there are still many challenges that remain to be addressed. The range of catalytic functionalities of nanozymes need to be further expanded to other enzyme classes, and the catalytic activity and specificity of nanozymes need to be improved. In contrast to natural enzymes that are typically highly selective not only for specific reactions but also to a single or selected few substrates; nanozymes tend to show a much wider substrate scope, and in certain cases multiple enzyme-like activities. Deciphering the mechanism of catalytic action of nanozymes will help us to better understand these differences. The nanozyme community needs to query the distinctness of nanozymes that set them apart from catalysts and bring them a step closer to the natural enzymes. Until these aspects are clearly addressed, some in the community are likely to see the rapidly emerging field of nanozymes with an iota of apprehension. Irrespective of these scrutinies and fundamental questions whether “nanozyme” is a true enzyme-mimic, it remains beyond a doubt that these types of materials offer remarkable potential for a variety of applications. In the context of biomedical applications of nanozymes, more efforts are needed to balance the surface modification strategies and optimum enzymatic activity of nanozymes with an emphasis to regulate the multi-enzymatic activities of nanozymes. In addition, the safety concerns of nanozymes for *in vivo* applications also need to be managed, as is a common practice with any foreign material introduced in the body.

We hope that this special issue will be able to share the latest advances in the field, provide current investigators with some food for thought, and inspire researchers with sparkling ideas to further advance the field of nanozymes. Last, but not the least, we would like to thank all the authors, reviewers, and the Frontiers in Chemistry development team for their great efforts in producing this special issue.

## Author Contributions

KF conceived and drafted the manuscript. YL and VB discussed and commented on the manuscript. All authors contributed to the article and approved the submitted version.

## Conflict of Interest

The authors declare that the research was conducted in the absence of any commercial or financial relationships that could be construed as a potential conflict of interest.

